# Adherence to antiretroviral therapy among people living with HIV/AIDS in northeastern Brazil: a cross-sectional study

**DOI:** 10.1590/1516-3180.2019.0212170919

**Published:** 2020-03-06

**Authors:** Rita de Cassia Albuquerque Soares, Ana Maria de Brito, Kledoaldo Lima, Tiago Maria Lapa

**Affiliations:** I MSc. Doctoral Student, Hospital das Clínicas, Universidade Federal de Pernambuco (UFPE), Recife (PE), Brazil.; II PhD. Researcher, Instituto Aggeu Magalhães, Fundação Oswaldo Cruz (FIOCRUZ), Recife (PE), Brazil.; III PhD. Laboratory Analyst (Biomedicine), Hospital das Clínicas (HC), Universidade Federal de Pernambuco (UFPE), Recife (PE), Brazil.; IV PhD. Researcher, Instituto Aggeu Magalhães, Fundação Oswaldo Cruz (FIOCRUZ), Recife (PE), Brazil.

**Keywords:** HIV, Antiretroviral therapy, highly active, Epidemiology, Brazil, Pharmacology, Adherence, People living with HIV/AIDS, AIDS, Nonadherence.

## Abstract

**BACKGROUND:**

Nonadherence to antiretroviral therapy (ART) may lead to viral replication and development of antiretroviral resistance.

**OBJECTIVE:**

To identify the factors associated with nonadherence to ART among people living with the human immunodeficiency virus (HIV)/acquired immunodeficiency syndrome (AIDS) (PLWHA).

**DESIGN AND SETTING:**

Cross-sectional study in a tertiary-level hospital in northeastern Brazil.

**METHODS:**

Intake of less than 90% of the antiretroviral drugs prescribed in the last week prior to the interview was defined as nonadherence. Intake was evaluated using a questionnaire. Descriptive and multivariate analyses were conducted on the study population, with estimation of the respective odds ratios and 95% confidence intervals.

**RESULTS:**

The prevalence of nonadherence was 28.4%. Significant associations were found regarding the following variables: age less than 35 years, smoking, sedentary lifestyle, lack of medication and lack of knowledge regarding the patient’s HIV status, on the part of the patient’s partner or family.

**CONCLUSIONS:**

Encouragement of adherence to antiretroviral therapy is one of the fundamental pillars of treatment for HIV-infected patients. The high proportion of nonadherence (28.4%) and the predictive factors related to this indicate that it is necessary to improve patients’ adherence to antiretroviral therapy.

## INTRODUCTION

Antiretroviral therapy (ART) decreases the viral load of the human immunodeficiency virus (HIV) or even renders it undetectable.^1^^,^^2^ However, problems relating to adherence to this therapy are practically universal, and adherence is a complex process that requires a multifaceted approach for its improvement.^3^ Nonadherence to antiretroviral drugs may consequently lead to development of viral resistance, which results in increased viral replication and development of opportunistic infections and other diseases, thereby increasing the morbidity and mortality associated with HIV infection.^4^

The ART adherence rates in Brazil vary from 20% to 84%.^5^^,^^6^^,^^7^^,^^8^^,^^9^ However, the way in which this adherence to therapy is measured differs between studies. In a meta-analysis on ART adherence rates in Latin American and Caribbean countries, Costa et al.^10^ found that they varied according to the length of time over which the measurement was made. Thus, individuals’ adherence was found to be inversely proportional to the duration of the measurement period.

Data regarding adherence obtained through self-reporting has been identified as more reliable than data obtained through medical evaluation.^11^ Moreover, it has been found that its accuracy can be amplified through use of questions that are less direct and with absence of prejudgment.^12^

Several factors have been correlated with nonadherence to ART among HIV-infected patients. Pinheiro et al.^13^ found that elderly patients had a higher adherence rate and, consequently, that a higher proportion of these patients had an undetectable viral load. Additionally, nonadherence has been correlated with use of illicit drugs,^14^^,^^15^ smoking,^14^^,^^16^ alcohol consumption,^16^ depressive symptoms,^17^ lower schooling levels and economic status,^8^^,^^15^ adverse reactions,^18^ symptoms of opportunistic diseases^8^ and longer time between infection and receiving the diagnosis of HIV and starting treatment.^15^

Interruption or irregularity of use of antiretroviral drugs is a public health problem in the fight against the HIV epidemic. Better adherence to therapy among patients not only leads to virological failure but also reduces HIV resistance to antiretrovirals (ARVs).^19^ Although several studies have been conducted in Brazil regarding ART adherence in the northeastern region of this country, more data is still needed, in order to broaden the notions about this issue and, thus, to enable creation of strategies elaborated on the basis of acquired knowledge.

## OBJECTIVES

The aim of this study was to identify the predictive factors that might define the profile of individuals at the highest risk of interruption of treatment, at a large HIV/AIDS treatment center in the northeastern region of Brazil.

## METHODS

### Study design and population

This was a cross-sectional study that investigated nonadherence to antiretroviral treatment among people living with HIV/AIDS (PLWHA) who were treated at the infectious and parasitic diseases outpatient clinic of a tertiary-level hospital (Hospital das Clínicas, Federal University of Pernambuco, Brazil) in the northeastern region of Brazil between November 2012 and March 2013.

The study population consisted of PLWHA who had been diagnosed in accordance with the following criteria used by the Brazilian Ministry of Health:^20^ individuals with a prescription for ART, aged 18 years or older, without mental illness, and who accepted participation in this study through signing a free and informed consent statement. A total of 253 people were interviewed.

### Adherence to antiretroviral therapy and variables analyzed

The interview data were recorded in a questionnaire that had been elaborated in a previous study.^5^ Nonadherence was defined as intake of less than 90% of the medications prescribed in the last week, considering the number of pills taken by asking the patients and the timetable for each intake.^5^^,^^21^

The outcome variable of this study was characterized as nonadherence in the week prior to the interview (self-reported). The independent variables were of three types, as follows:

- Sociodemographic variables and social behavior: region of residence, sex, age, schooling level, marital status, occupation, race, alcohol consumption, smoking, illicit drug use, religion and physical activity (defined as a minimum 30 minutes, three times a week);
- Variables relating to epidemiological data and support network: HIV transmission route, duration of infection, gender, stable or long-term relationship, number of stable or long-term partners, having casual partners, number of casual partners, HIV-infected partners, partners’ knowledge of the subject’s HIV status, condom use before HIV infection, use of condoms after HIV diagnosis, family knowledge of the subject’s HIV status, family support, support from friends/partners and level of knowledge about HIV/AIDS;
- Variables relating to antiretroviral therapy and pharmaceutical and medical care: duration of use of ART; current ARV scheme; reason for stopping taking ARVs; difficulty in obtaining the ARV from the dispensing pharmacist and what this difficulty was; difficulty in medical consultations and what this difficulty was; any other chronic illness; whether other medicines were being taken constantly and what these medicines were; whether the doctor had given explanations regarding the subject’s health condition; whether the subject felt informed about HIV/AIDS; whether the subject talked to his/her doctor about his/her health condition; the subject’s knowledge of ARV, including the names of ARVs; whether the subject’s doctor talked about the ARV medications used; whether the pharmacy talked about the subject’s use of ARV medicines; whether the subject felt secure about how to take his/her medications; and whether the subject would, if he/she had questions about the medicines, ask the doctor, the pharmacist or another professional.

### Statistical analyses

The prevalence of nonadherence and the statistical association between the variables and the outcome were investigated using the chi-square test through the Epi Info software, version 3.5.3. P < 0.05 was considered statistically significant. For multivariate analysis, logistic regression was performed. The variables included were those that showed statistical significance in the bivariate analysis. The odds ratio, confidence interval and likelihood were calculated for each variable, with the respective significance tests. Regression analysis was performed using SPSS version 10.0.

### Ethical considerations

The present work was approved, in accordance with the norms of Resolution 196/96 of the National Health Council, which regulates research activities on human beings, by the research ethics committees of Aggeu Magalhães Research Center, under the number 04911912.5.0000.5190, on September 5, 2012.

## RESULTS

Among the 253 PLWHA who were assessed, 28.4% (n = 72) demonstrated nonadherence to ARVs. Among these individuals, 65% were men, 78% were over 35 years old, 78% practiced some type of religion, 58% had long-term partners and 26% had not informed their partners about living with HIV/AIDS. Nonadherence to ART was statistically associated with younger age, smoking, use of illicit drugs and with having some religion or practicing physical activity (Table 1).

**Table 1.** Sociodemographic characteristics and social behavior of adults with human immunodeficiency virus (HIV) infection, in relation to nonadherence to treatment with antiretrovirals

Variables	Nonadherence		Adherence		χ^2^*	P-value
	n = 72	%	n = 181	%		
Age (years)						
18-35	23	31.9	33	18.3	0.17	0.01
> 35	49	68.0	148	81.7		
Consumption of alcoholic beverage on a public holiday (glasses)						
1 to 12	30	66.7	66	80.5	3.01	0.08
> 13	15	33.3	16	19.5		
Smoking						
Yes	22	30.5	31	17.2	5.60	0.01
No	50	69.5	150	82.8		
Illicit drugs						
Yes	9	12.5	7	16.3	6.48	0.01
No	63	87.5	174	96.7		
Religious activity						
Yes	49	68.1	149	82.3	6.16	0.01
No	23	31.9	32	17.7		
Physical activity						
Yes	20	27.8	80	47.1	7.75	0.005
No	52	72.2	90	52.9		

*χ^2^ = chi-square test.

It was found that 32% and 38% of the nonadherent and adherent individuals, respectively, reported that there was a lack of antiretroviral medication. Furthermore, 30% of the nonadherent individuals had had some type of difficulty in obtaining their medications from the dispensing pharmacist and that they had had greater difficulties in making new appointments for medical consultations than the adherent individuals, thus revealing lapses in medical and pharmaceutical care.

Another important point was that adherent individuals reported receiving better information about ARVs from the attending physicians, which may have directly influenced the therapeutic results (Table 2).

**Table 2.** Characteristics of antiretroviral therapy and medical and pharmaceutical care, in relation to nonadherence to antiretroviral therapy

Variables	Nonadherence		Adherence		χ^2^*	P-value
	n = 72	%	n = 181	%		
Antiretroviral (ARV) schemes						
2 NRTI + NNRTI	29	40.3	100	55.0	4.61	0.03
Others	43	59.7	81	45.0		
Reason for stopping taking ARVs						
Lack of medication	23	31.9	49	37.6	3.57	0.05
No reason/depression/forgetfulness	49	68.1	81	62.4		
Difficulty in obtaining medicines						
Yes	21	29.2	34	18.8	3.26	0.07
No	51	70.8	147	81.2		
Difficulty to getting a consultation with a doctor						
Yes	21	29.2	31	17.1	4.57	0.03
No	51	70.8	150	82.9		
Chronic illness						
Yes	13	18.3	51	28.2	2.62	0.10
No	58	81.7	130	71.8		
Do you use other medicines constantly?						
Yes	13	18.1	50	27.8	2.59	0.10
No	59	81.9	130	72.2		
Do you talk to your doctor about your medicines?						
Yes	30	41.7	93	51.4	1.95	0.16
No	42	58.3	88	48.6		
Does your doctor talk to you about your medications?						
Yes	45	62.5	137	75.7	4.44	0.03
No	27	37.5	44	24.3		
Do you think you should be more involved in decisions about your treatment?						
Yes	51	70.8	110	61.1	2.11	0.14
No	21	29.2	70	38.9		

*χ^2^ = chi-square test.

NNRTIs = non-nucleoside reverse transcriptase inhibitors; NRTIs = nucleoside reverse transcriptase inhibitors.

Regarding epidemiological characteristics, nonadherent patients had a higher frequency of casual partnerships and their partners and families were not aware of their HIV status (Table 3).

**Table 3.** Epidemiological and supportive network characteristics of adults with human immunodeficiency virus (HIV) infection, in relation to nonadherence to antiretroviral therapy

Variables	Nonadherence		Adherence		χ^2^*	P-value
	n = 72	%	n = 181	%		
Long-term partners						
Yes	49	69.1	98	57.9	2.56	0.10
No	22	30.9	71	42.1		
Casual partners over the last 12 months						
Yes	30	41.7	45	26.4	5.46	0.01
No	42	58.3	125	73.6		
Number of casual partners over the last 12 months						
1 to 9	23	76.6	42	89.4	2.24	0.13
≥ 10	7	23.3	5	10.6		
Partner infected with HIV						
Yes	26	41.9	74	60.5	5.81	0.01
Unknown HIV serological status	36	58.1	48	39.5		
Does your partner know about your serological status (HIV)?						
Yes	33	54.1	89	70.6	4.96	0.02
No	28	45.9	37	29.4		
Have you have spoken to someone in your family about your HIV status?						
Yes	49	72.1	153	85.0	5.47	0.01
No	19	27.9	27	15.0		

*χ^2^ = chi-square test.

The multivariate model revealed that the variables associated with nonadherence were younger age, smoking, sedentary lifestyle, lack of medication and alienation of the subject’s partner and family regarding his/her HIV serological status (Table 4).

**Table 4.** Multivariate analysis on associations with nonadherence to antiretroviral therapy among human immunodeficiency virus (HIV)-positive patients

Variables	OR	95% CI	P-value*
Age (years)			
18 to 35	2.105	(1.010-4.385)	0.047
> 35	1.0		
Smoking			
Yes	2.168	(1.046-4.491)	0.037
No	1.0		
Physical activity			
Yes	1.0	(1.059-4.085)	0.033
No	2.080		
Does your partner know about your HIV status?			
Yes	1.0	(1.278-4.927)	0.008
No	2.510		
Does your family know about your HIV status?			
Yes	1.0	(1.179-5.651)	0.018
No	2.581		
Lack of antiretrovirals			
Yes	2.434	(1.285-4.609)	0.006
No	1.0		

OR = odds ratio; CI = confidence interval; *χ^2^ = chi-square test.

## DISCUSSION

The rate of nonadherence detected among HIV-infected individuals undergoing ART at the Department of Infectious Diseases of Hospital das Clínicas, Federal University of Pernambuco, in the northeastern region of Brazil, was 28.4%. The multivariate analysis showed that there were statistical associations between nonadherence to ART and younger individuals, smokers, individuals with sedentary lifestyle, individuals whose partners and/or family were unaware of their HIV status and individuals who reported a lack of medication during treatment. Other studies^22^^,^^23^ have also demonstrated an association between smoking and nonadherence. Regarding the association between nonadherence and younger individuals, there is a consensus in the literature that, in cases of chronic diseases, adherence increases with age.^24^^,^^25^ Brazilian studies have ratified this type of association among HIV-positive individuals of younger age.^13^^,^^15^

Adults living with HIV can expect to gain many benefits from aerobic exercise, with improved cardiorespiratory function and psychological health.^26^ Thus, the association between practicing exercise and adherence to therapy may be related to a possible improvement of psychological health, thereby leading to self-care. This conclusion has been confirmed through the observation that depressed patients or patients with other psychological disorders have less adherence to ARVs.^17^^,^^27^

The association between nonadherence and the variable of lack of knowledge among partners and relatives of the subject’s HIV serological status may be related to social isolation. This is a common phenomenon among HIV-infected individuals and is caused by fear of social stigma. Hence, maintaining confidentiality seems to be the most adequate solution.^28^ Self-imposed stigma can result in exclusion from social life and stable sexual relationships. Social support plays an important role in mitigating the negative consequences of stressful events, while insufficient support from people in the social and familial environment seems to negatively affect adherence.^29^

Individuals with greater difficulties in consulting their attending physicians and those who reported experiencing a lack of medications had higher nonadherence rates. Thus, it was seen that inaccessibility of healthcare services was a predictive factor for lack of correct adherence to ART. Additionally, approximately 32% of the nonadherent and 38% of the adherent patients reported experiencing a lack of medication at some time during their treatment. On the other hand, adherent individuals reported obtaining more information about ART from their attending physicians, which may have correlated with better treatment.

Thus, the role of the healthcare services is extremely important in relation to adherence. If dialogue with and communication from the service are available, a more reliable and committed relationship becomes possible. This broadens the understanding of adherence and, because of its multiple dimensions, a new adherence paradigm emerges. This consists of a model that is built between the patient, the healthcare team and the healthcare service.

One limitation of the present study relates to its use of information that only came from self-reports, although a check on the therapeutic schemes used was made available through the logistic control system for medicines (Sistema de Controle Logístico de Medicamentos, SICLOM) of the Brazilian Ministry of Health. For future studies, it is recommended that self-reporting should be confirmed through patient records, although this does not always address important issues regarding drug adherence.

Another limitation of the present study may have been its selection criterion, given that participation bias was present because the sampling was done according to convenience.

Encouragement of adherence to ART is one of the fundamental pillars of treatment for HIV-infected patients. Adequate adherence results in virological suppression, increased CD4+ T-cell counts and decreased morbidity and mortality. Additionally, nonadherence to ART is related to virological failure and antiretroviral resistance, with possible dispersion of resistant viruses. Therefore, working to promote adherence is also an important preventive tool within public health.^19^

Because of the policy of universal access to antiretroviral drugs in Brazil, the topic of adherence has been widely discussed in this country. However, there continues to be a lack of reports from the northeastern region, where the HIV epidemic is on the rise. Hence, studies on adherence to antiretroviral treatment remain extremely important, to enable better understanding of the problem and ensure adequate performance among multiprofessional teams.

## CONCLUSIONS

This study showed that the prevalence of nonadherence was 28.4% and that the following factors presented statistical associations with it: age from 18 to 35 years, smoking, sedentary lifestyle, lack of knowledge among partners and families regarding the patient’s serological status, lack of medication and difficulty in consulting a doctor. Identification of factors that are predictive of nonadherence will allow the multidisciplinary teams caring for PLWHA to adopt measures that increase drug adherence and will contribute towards establishment of information for a surveillance system.
